# Interventions Targeting HIV-Infected Risky Drinkers: Drops in the Bottle

**Published:** 2010

**Authors:** Jeffrey H. Samet, Alexander Y. Walley

**Keywords:** Alcohol and other drug use, alcohol consumption, alcohol use disorder, human immunodeficiency virus, HIV-infected patients, sexually transmitted disease, unsafe sex, treatment method, treatment outcome, intervention, clinical trial, literature review

## Abstract

Alcohol use is common among people infected with HIV and may contribute to adverse consequences such as reduced adherence to treatment regimens and increased likelihood of risky sexual behaviors. Therefore, researchers and clinicians are looking for treatment approaches to reduce harmful alcohol consumption in this population. However, clinical trials of existing treatment models are scarce. A literature review identified only 11 studies that included HIV-infected patients with past or current risky alcohol use and which targeted alcohol use and other health behaviors. Four studies focusing on HIV-infected participants with alcohol problems found mixed effects on adherence and on alcohol use. Five clinical trials included at least 10 percent of HIV-infected subjects who use alcohol; of these, only one reported significant evidence of a favorable impact on alcohol consumption. Finally, two trials targeting alcohol users at high risk for HIV infection identified treatment effects that were not sustained. Taken together, these findings provide limited evidence of the benefit of behavioral interventions in this population. Nevertheless, these studies give some guidance for future interventions in HIV-infected patients with alcohol problems.

In the United States, people infected with the human immunodeficiency virus (HIV) drink more alcohol than people in the general population. Specifically, a higher proportion drink risky amounts^1^ or have an alcohol use disorder (i.e., abuse or dependence) (Conigliaro et al. 2003; Galvan et al. 2002; Lefevre et al. 1995; Samet et al. 2003*a*,*b*, 2004). Risky alcohol use in HIV-infected people has been associated with the following range of adverse effects:

- Reduced adherence to medication regimens for treatment of HIV infection (Chander et al. 2006; Conen et al. 2009; Cook et al. 2001; Golin et al. 2002; Halkitis et al. 2003; Samet et al. 2004);
- Lack of a health care provider for the HIV infection (Metsch et al. 2009);
- Delayed linkage to HIV medical care (Samet et al. 1998);
- Increase in risky sexual behaviors (Kalichman et al. 2002; Metsch et al. 2009);
- Increased transmission of sexually transmitted infections (Kalichman et al. 2000); and
- Progression of HIV disease (Conigliaro et al. 2003; Miguez et al. 2003; Samet et al. 2007).

Given the spectrum of problems associated with such alcohol use among HIV-infected patients, one important avenue to improving the health of this population is to develop interventions that target alcohol use and its associated consequences. Accordingly, interventions have been designed to both decrease alcohol consumption and address the specific adverse health consequences.

The concept that negative consequences of alcohol use can be reduced in patients with HIV infection is based on research demonstrating the impact of clinical interventions on alcohol consumption and associated negative consequences in patients without HIV infection (Institute of Medicine 1990; Kristenson et al. 1983). Alcohol research over the past three decades has demonstrated that behavioral interventions can be effective, with benefits varying based on setting, severity of alcohol problems, and patient characteristics. For example, meta-analyses of randomized controlled trials (RCTs)^2^ of interventions to reduce risky alcohol use demonstrated decreased drinking for patients in primary care settings (Beich et al. 2003; Kaner et al. 2007). However, no such effects were found in meta-analyses of interventions delivered in hospital settings (Emmen et al. 2004), possibly because inpatients typically have greater severity of alcohol problems (i.e., most are alcohol dependent) (Saitz et al. 2007, 2008). Several high-quality RCTs of brief interventions delivered in emergency departments also detected no or limited benefit (D’Onofrio and Degutis 2002; Daeppen et al. 2007; Longabaugh et al. 2001; Monti et al. 1999). The influence of the patient’s consumption levels also was demonstrated in several studies. For example, in two separate RCTs in the primary-care setting (Fleming et al. 1997; Ockene et al. 1999), where patients were seeking medical care but not necessarily for an alcohol problem, implementation of a 5- to 15-minute discussion reduced alcohol consumption in patients who met the criteria for risky drinking. Studies of such brief interventions among patients who met the criteria for alcohol dependence, however, have shown no benefit (Kaner et al. 2007; Whitlock et al. 2004; Wutzke et al. 2002).

For alcohol-dependent patients, more extensive behavioral interventions (e.g., cognitive–behavioral coping skills, motivational enhancement, 12-step facilitation) can be effective *Project* (*MATCH* Research Group 1997). In addition, several medications (i.e., disulfiram, naltrexone, and acamprosate) are approved for the treatment of alcohol dependence, and other medications (e.g., topiramate) are being further evaluated (Anton et al. 2006; Garbutt et al. 2005; Kranzler and Van Kirk 2001; Olmsted and Kockler 2008; Rubio et al. 2001).

Given the strong evidence that alcohol consumption is an important health issue for many people with HIV infection, efforts to potentially ameliorate these problems by addressing alcohol use are of great interest. The studies in non–HIV-infected people reviewed above suggest that interventions among HIV-infected people with alcohol problems could be beneficial. However, the wide range of results in these intervention studies based on setting and disease severity argues for the need to carefully assess efforts to mitigate alcohol’s deleterious impact on health in HIV-infected patients. As an important step in this direction, this article summarizes the findings of a review of the clinical trial literature on interventions addressing alcohol consumption and its consequences among HIV-infected patients. After describing the design of the literature search and evaluation, the article reviews the findings of the studies identified and discusses the implications of those findings.

## Design of the Literature Review

The literature review sought to identify clinical trials of interventions among HIV-infected people with past or current unhealthy alcohol use (i.e., the spectrum from risky drinking to alcohol dependence [Saitz 2005]) that reported effects on any of the following outcomes:

- HIV disease progression;
- Receipt of HIV treatment;
- HIV medication adherence;
- HIV risk behaviors;
- Acquisition of sexually transmitted infections; and
- Alcohol use.

To be included in the review, the studies had to report alcohol-specific outcomes. Beyond that, the studies were classified into three categories of specificity. The most specific category comprised clinical trials that included only HIV-infected people with past or current unhealthy alcohol use. The second category comprised clinical trials that included only HIV-infected people but in which not all of the participants exhibited unhealthy alcohol use. For a study to be included in this category, at least 10 percent of participants had to report current alcohol use. The third category of studies comprised trials that were aimed at preventing alcohol use and sexual behaviors that put people at risk of HIV infection among alcohol-using people. Although these studies did not include HIV-infected participants or did not report the HIV status of the participants, they were reviewed because they may inform future research on people at risk of HIV transmission in the setting of alcohol use.

Initially, the review intended to include only RCTs. However, very few studies were identified that met this criterion in the first two categories. Therefore, the search was expanded to include nonrandomized and non-controlled clinical intervention trials in categories 1 and 2.

To identify relevant studies, the literature database MEDLINE was searched through September 30, 2009, using the search terms “HIV, alcohol, hazardous drinking, risky drinking, problem drinking, counseling, brief intervention, 12 step, pharmacotherapy, naltrexone, acamprosate, disulfiram, topiramate, and clinical trial.” For all articles identified using this approach, the reference lists also were scanned, as were related articles identified by the search engine for the MEDLINE data base to look for additional studies. Reference lists for articles that were closely related, but did not meet the criteria, also were reviewed. Finally, articles referenced in relevant review articles were examined. Titles of all articles were reviewed to determine if the articles met the selection criteria. If the nature of the study could not be discerned through the title, the abstract and/or full text of the article was retrieved and reviewed.

For all studies that met the criteria for one of the three categories, information on the setting, study design, methodological quality, type of intervention, outcomes reported, period of follow-up, and results was extracted. The following sections summarize the findings of these analyses. They are presented as a descriptive narrative synthesis because studies were too few and heterogeneous to perform a standard meta-analysis.

## Results of the Literature Review

The search strategy described above identified 241 potentially relevant studies that were evaluated further. Of these, four studies including a total of 578 patients (Aharonovich et al. 2006; Parsons et al. 2007; Samet et al. 2005; Velasquez et al. 2009) met the selection criteria for the first category (see table 1). Another five clinical trials that included 1,311 patients (Gilbert et al. 2008; Naar-King et al. 2006, 2008; Rotheram-Borus et al. 2001, 2009; Sorensen et al. 2003) fell into the second category. In addition, two informative studies of interventions among people at high-risk for HIV reported outcomes specific to alcohol use (Kalichman et al. 2008; Morgenstern et al. 2007). All of these studies are reviewed below. Some other studies that involved alcohol-using, HIV-infected patients, but were excluded from this discussion because of serious design or methodological limitations, are listed in Table 2 because they may inform additional research. Interestingly, no controlled trials of the four medications recommended by NIAAA (2007) for the treatment of alcohol dependence (i.e., disulfiram, naltrexone, acamprosate, and topiramate) have been conducted in HIV-infected patients.

## Clinical Trials Among HIV-Infected People With Past or Current Unhealthy Alcohol Use

### Velasquez and Colleagues (2009) Study

These investigators conducted an RCT among 253 HIV-infected men who had had sex with men in the previous 3 months and who scored more than eight points on the AUDIT questionnaire (Babor et al. 2001). The intervention group received four manual-guided individual sessions and four manual-guided peer education and support group sessions that utilized motivational interviewing (MI) counseling strategies (Miller and Rollnick 2002) to guide participants through the stages of change of Prochaska and DiClemente’s Trans-Theoretical Model^3^ (Prochaska and DiClemente 1982). In contrast, the control group received educational materials on HIV and alcohol, referral information, and advice to stop or cut back on their alcohol use. At the 12-month follow-up, the investigators determined some benefits of the intervention on some of the measures evaluated. For example, the control group had 1.4 times the number of drinks per 30 days and 1.5 times the number of heavy-drinking days per 30 days compared with the intervention group. For other measures (e.g., having anal sex without a condom, number of drinking days, or number of days on which both drinking and sex occurred), however, no significant difference existed between the two groups. Only when the analysis of same-day drinking and sex was restricted to participants who had shown this behavior at baseline, did those in the control group have significantly (i.e., 2.19 times) more days on which drinking and sex occurred than the intervention group. The interpretation of these findings is limited by the fact that there was differential loss to follow-up—that is, the analyses included only 81 percent of participants randomized to the intervention group and 90 percent of subjects randomized to the control group. Thus, one cannot exclude the possibility that particularly in the intervention group, participants with worse outcomes were not included in the analysis.

### Aharonovich and Colleagues (2006) Study

In this pilot study, 31 HIV-infected primary-care patients with heavy alcohol use received one session of MI from a trained counselor, followed by daily telephone-based interactive voice response (IVR) assessments of drinking amounts and graphic feedback of changes in drinking at 30 and 60 days. This intervention resulted in a decrease in the number of drinks per day at 30 and 60 days (from 3.2 drinks per day at baseline to 1.7 drinks at 30 days and 1.2 drinks at 60 days). The IVR system was utilized; 77 percent of all possible daily calls were completed at 30 days. However, these improvements can not be attributed to the intervention with confidence because there was no control group.

### Parsons and Colleagues (2007) Study

These investigators conducted an RCT among 143 HIV-infected people with “hazardous drinking” (defined as more than 16 standard drinks per week for men or more than 12 standard drinks per week for women), assessing treatment effects on HIV medication adherence and alcohol outcomes. The intervention involved eight 1-hour individual sessions of MI and cognitive behavioral skills training over 3 months and was compared with a time- and content-equivalent control.^4^ Over the follow-up period (3 and 6 months), both groups exhibited substantial improvement for both total alcohol drinks over 14 days or drinks per drinking day, although no significant differences existed between the intervention and the control group. However, compared with the control group, the intervention did improve medication adherence, number of virus particles detectable in the blood (i.e., viral load), and CD4 cell ^5^ counts at 3 months. These statistically significant improvements were not sustained at 6 months.

### Samet and Colleagues (2005) Study

This RCT included 151 HIV-infected patients on antiretroviral therapy (ART) who had a history of alcohol problems. The participants received either four nurse-delivered, 30- to 60-minute sessions focusing on HIV medication adherence and alcohol counseling, both in a clinic and at home, or no intervention. The study found no significant differences between groups upon examination of the following outcomes: 3-day medication adherence, 30-day adherence, CD4 cell count, viral load, drinks per day, percent reporting drinking, or percent reporting hazardous drinking. Study limitations were that not all participants were non-adherent to their HIV medication at baseline and a substantial percentage were not in the risky-drinking range of unhealthy alcohol use, the group most amenable to brief interventions.

## Clinical Trials Among HIV-Infected People of Whom at Least 10 Percent Currently Use Alcohol

Five studies identified in the literature review fell into this category, and only one of these (Rotheram-Borus et al. 2009) demonstrated significant treatment effects on alcohol use (see table 1). This study was a subanalysis of a parent RCT among 936 HIV-infected people who were sexually active without a condom with at least one HIV-negative partner or two HIV-infected partners (Wong et al. 2008). The participants received either 15 90-minute individual sessions of cognitive–behavioral therapy (CBT) delivered over 15 months or usual care. The subanalysis by Rotheram-Borus and colleagues (2009) was limited to 270 HIV-infected participants who were homeless or without stable housing. In this group, the intervention was found to reduce alcohol or marijuana use from 36 to 28 days in the prior 90 days, whereas in the control group the frequency of alcohol or marijuana use was unchanged at 35 of the last 90 days. However, this study had substantial methodological limitations, some of which pertain to the parent study. For example, in the parent study, random assignment of participants to the groups resulted in an imbalance between the groups with respect to baseline HIV risk behaviors or demographics. Moreover, the sub-analysis was limited to participants who completed four follow-ups and were homeless or without stable housing. Finally, the outcome was alcohol or marijuana use in the last 3 months with no alcohol-specific results provided.

The four other studies in this category did not demonstrate any significant effects of the interventions tested on alcohol use:

- In a preliminary analysis of 3-month outcomes among 51 subjects randomized to four 1-hour motivational enhancement therapy sessions in an adolescent clinic, Naar-King and colleagues (2006) observed a trend, but no statistically significant reduction, in the number of drinks per week during the week with the maximum number of drinks. Moreover, in the final analysis of the study, which included 65 subjects, 39 percent of whom used alcohol, this difference was not sustained at 6 or 9 months (Naar-King et al. 2008).
- Gilbert and colleagues (2008) randomized 476 HIV-infected patients, 38 percent of whom reported risky drinking, to an MI-based “Video Doctor” intervention via laptop computer or a control group receiving usual care. The intervention resulted in decreased 30-day illicit drug use, lower mean number of drug use days, and a modest reduction of unprotected sex at 3 and 6 months. However, no differences in alcohol use existed between the intervention and control groups.
- Sorensen and colleagues (2003) randomly assigned HIV-infected patients with drug dependence, 61 percent of whom reported current alcohol use, to 1 year of continuous case management or to a brief contact (i.e., one HIV risk education session and printed information). No differences were noted in alcohol outcomes at 6, 12, or 18 months.
- A study among HIV-infected youths compared the effects of 23 2-hour group sessions and usual care on risk behaviors (Rotheram-Borus et al. 2001). The investigators found no changes from baseline on a measure reflecting alcohol and marijuana use and no difference between the intervention and control groups.

## RCTs Among Alcohol Users at High-Risk for HIV Infection

Two informative RCTs have been conducted among alcohol drinkers at high risk for HIV infection. Morgenstern and colleagues (2007) performed a study with 198 high-risk, HIV-negative men who had sex with men and who were diagnosed with alcohol abuse or dependence but were seeking to moderate their alcohol use. The investigators compared the effects of 12 weekly MI sessions augmented with CBT with 4 sessions of MI alone. Unexpectedly, the investigators found that the nonaugmented MI group had less drinking and fewer alcohol-related drinking problems than the MI-plus-CBT group during the 12 weeks of the intervention and that there were no significant differences at 12-month follow-up. Thus, the addition of CBT to MI techniques provided no additional benefit regarding alcohol outcomes and potentially even diminished effects in this population. Subgroup analyses demonstrated that the detrimental effect of augmentation occurred particularly in participants with a concomitant drug use disorder.

Another RCT (Kalichman et al. 2008) compared a 3-hour, skills-based HIV and alcohol risk reduction group session with a 1-hour HIV/alcohol information group session among 342 South Africans frequenting drinking establishments. In this study, the extended session resulted in decreases in alcohol use before sex and unprotected intercourse at 3 month but not at 6 month follow-up. Moreover, intervention effects were stronger in participants drinking less at baseline.

## Discussion

Given the high prevalence of unhealthy alcohol use among HIV-infected people and its associated adverse health consequences, development of clinical and public health interventions that seek to address alcohol use and improve health outcomes in this population is a priority. In recognition of this, NIAAA, as early as 1996, issued a request for applications entitled “Developing Alcohol-Related HIV Preventive Interventions (AA–97–03).” Since then, several studies have been published that describe clinical outcomes of interventions in this population. However, as this article has demonstrated, the literature on this important topic still is not extensive. A literature search revealed only four clinical intervention studies focusing exclusively on HIV-infected patients with current or past unhealthy alcohol use; five other clinical trials included and documented the alcohol use of some of their HIV-infected participants. Overall, the current state of research strongly suggests that although the problems related to alcohol in HIV-infected people are abundant, effective interventions are few and new ones are urgently needed. Hence, addressing alcohol problems remains an important issue in HIV research.

Not only are studies among alcohol-abusing, HIV-infected patients scarce, but the existing studies also yielded mixed results. Two of the four studies that specifically targeted HIV-infected people with alcohol problems showed improvement in drinking outcomes. Velasquez and colleagues (2009) demonstrated reduced drinking levels over 12 months after an intervention that included both MI and peer support. The intervention was particularly strong in reducing same-day drinking and sex, which compels further research on interventions targeting alcohol use at the time of HIV risk behaviors (Velasquez et al. 2009). Although the intervention types used in the study only were shown to be effective in a sample of men who have sex with men, they warrant study among other populations. In the other study, Aharanovich and colleagues (2006) demonstrated the feasibility of ongoing telephone-based interactive voice response and graphic feedback, which should inspire the inclusion of automated, tailored, ongoing intervention boosting as part of behavioral interventions. It is important to note, however, that both these studies had methodological limitations (e.g., substantial or differential loss to follow-up, incomplete assessments) and their findings therefore are not definitive. Nevertheless, they provide some guidance for future more rigorous clinical trials.

The other two clinical trials (Parsons et al. 2007; Samet et al. 2005) among alcohol-abusing HIV-infected people attempted to improve ART adherence. This is an appropriate target of alcohol intervention studies in this population because medication adherence is of utmost importance for achieving good HIV disease outcomes, and alcohol-using patients have been documented to exhibit suboptimal ART adherence (Braithwaite et al. 2005; Chander et al. 2006; Conen et al. 2009; Samet et al. 2004). The results of both of these trials are discouraging, however, because although they explicitly addressed both alcohol use and medication adherence, one study (Samet et al. 2005) found no impact on adherence, alcohol consumption, or any HIV outcome, and the other (Parsons et al. 2007) only detected short-lived improvements (i.e., they were evident at 3 months, but not at 6 months). Thus, these two high-quality studies suggest that achieving clinically beneficial outcomes in HIV-infected people with alcohol problems is more difficult than has been the case with populations of HIV-infected without diagnosed unhealthy alcohol use (Amico et al. 2006; Simoni et al. 2006). Among the latter group, RCTs to improve adherence that used interventions with a range of intensities did reveal improvements in adherence which were sustained for up to 12 months, as well as in HIV viral load and CD4 counts (Tuldra et al. 2000). The difficulty of achieving positive benefits (e.g., improved ART adherence) through interventions among HIV-infected people who have alcohol problems also is evidenced by the study by Kalichman and colleagues (2008) among drinkers who were not infected with HIV. The findings of that study suggest that, as in brief intervention studies, intervention effectiveness varies by severity of alcohol use, with less improvement noted in dependent than in nondependent drinkers. Thus, levels of alcohol consumption, alcohol use disorder severity, and alcohol-related consequences are important covariates to be assessed and reported in HIV intervention studies.

A notable finding of this literature review was that as of 2009, no study of pharmacotherapy for alcohol dependence in HIV-infected patients had been published. This is surprising given that pharmacotherapy plays a major role in addressing the AIDS epidemic by improving outcomes of HIV-infected subjects. Moreover, some preclinical research has demonstrated that naltrexone, an effective medication for alcohol dependence, inhibits alcohol-mediated enhancement of HIV infection (Wang et al. 2006) and may potentiate the anti-HIV effects of antiretroviral medications (Gekker et al. 2001). Therefore, testing the effectiveness of naltrexone and other medications in alcohol-dependent HIV-infected patients is an important current research direction.

Two of the studies reviewed here that included HIV-infected patients among whom at least 10 percent currently used alcohol, targeted risky sexual behaviors rather than alcohol consumption. Assessing treatment effects on sex risk factors is appropriate for studies among HIV-infected drinkers because several studies have demonstrated an association between alcohol use and risky sex (Purcell et al. 2001; Stein et al. 2009). In both the study by Gilbert and colleagues (2008) and the study by Naar-King and colleagues (2006, 2008), sex risk behaviors were decreased in the group randomized to the intervention at 3 and 6 months, but there were no or only transient effects on alcohol use. These findings suggest that behavioral interventions which are not specifically tailored to address alcohol use are unlikely to impact alcohol problems in a sustained fashion.

The dearth of studies focusing on alcohol consumption among HIV-infected people is understandable. Although the spectrum of unhealthy alcohol use ranging from risky use to alcohol dependence occurs in this population, other pressing health concerns (e.g., ART adherence, risky sexual behaviors, or engagement in HIV care) appropriately become the main focus of clinical trials that also may address alcohol consumption in their intervention arms. Developing interventions that target a specific behavior (e.g., sex) at the time of alcohol use is a worthy pursuit, and understanding the importance of decreasing alcohol use in order to successfully achieve behavior change is crucial for developing future interventions.

One interesting development noted in the studies reviewed here was the use of new technology (e.g., interactive voice-response systems) in two of the studies (Aharonovich et al. 2006; Gilbert et al. 2008). These approaches to delivering a behavioral intervention merit further exploration because they have the potential for providing scalable, ongoing delivery of tailored automated messages that may boost a more intensive directly administered intervention.

When assessing the relevance of the studies reviewed here, particularly those conducted among HIV-infected patients with past or current unhealthy alcohol use, it is important to consider the methodological quality of the work (i.e., the potential for bias, design limitations, and outcome measures). The report by Velasquez and colleagues (2009) is the only controlled study demonstrating a sustained clinically significant treatment effect on an alcohol-specific outcome, making publication bias (i.e., the preferential publication of studies that find significant differences) unlikely.

Regarding their design, most, but not all, of these studies met important design criteria, such as random allocation of participants to treatment groups and intention-to-treat analyses^6^ in the presentation of results. As with all behavioral intervention studies, keeping participants in the dark about which treatment they receive (i.e., blinding of participants to their treatment) is not possible. However, both Parsons and colleagues (2007) and Gilbert and colleagues (2008) utilized time- and content-equivalent controls to allow for the detection of effects specific to the counseling method studied.

The outcome measures reported were not consistent across studies and not always meaningful, limiting the comparability of study outcomes. For example, Naar-King and colleagues (2006) used an alcohol-specific measure—the number of drinks per week during the week with the maximum number of drinks at 3 months—that is not widely used and of questionable clinical meaning. Sorensen and colleagues (2003) only report a measure called the Addiction Severity Index Alcohol Composite Score, without any explanation or reporting of the individual components, complicating judgment of its clinical meaning. Finally, Samet and colleagues (2005) focused on ART adherence as an outcome, yet this study may underestimate the effectiveness of the intervention because the criteria for eligibility to participate in the study did not exclude patients with already good adherence. Thus, participants with good adherence at baseline provided little opportunity for an intervention to reveal a clinically meaningful impact.

In summary, as of 2009 the medical literature on clinical trials focused on people with HIV infection and unhealthy alcohol use is limited (i.e., “drops in a bottle”). Few of these studies were able to document improved outcomes, and any effects observed generally were modest and transitory. Based on these findings and current knowledge, the following questions need to be addressed:

- What are the characteristics of interventions that mitigate the health consequences of alcohol use in HIV-infected people?
- How does the treatment setting impact the effectiveness of behavioral interventions?
- How can technology best be used to extend and enhance intervention effects?
- What characteristics of HIV-infected drinkers suggest greater challenges when attempting to improve clinical outcomes?
- How can individual, network, or community interventions in people with multiple overlapping problems, including alcohol use, optimally reduce unhealthy behaviors?
- How might combined pharma-cotherapy and behavioral therapy be utilized to address the spectrum of clinical consequences that accompany heavy alcohol consumption?

Obtaining answers to these questions is the key next step in the successful development of clinical and public health interventions to mitigate the adverse outcomes from alcohol use in HIV-infected patients.

## Figures and Tables

**Table 1:** Studies Identified During a Literature Search on Interventions to Decrease Alcohol Use and Related Behaviors among HIV-Infected People and Alcohol Users at High Risk for Infection

**Study**	**Population/Setting**	**Design**	**Outcomes/Results**	**Comments**
**Category 1: Clinical trials among HIV-infected people with past or current unhealthy alcohol use**				
Velasquez et al. 2009	**Population** : 253 HIV-infected men who had sex with men (MSM) in the previous 3 months and an AUDIT score of more than 8. **Setting**: Recruited from HIV organizations, advertising, and social venues between 1999 and 2003.	**Intervention:** Randomized Controlled Trial (RCT) of four sessions of motivational interviewing (MI)-based individual counseling and four sessions of transtheoretical model–based peer-group education/support. **Control:** HIV and alcohol educational materials, resource referrals, and advice to stop or reduce drinking. **Assessment:** Baseline, 3, 6, 9, and 12 months.	**Alcohol use:** Control group had 1.38 times the number of drinks per 30 days and 1.50 times the number of heavy drinking days per 30 days compared with the intervention group. **Sex risk:** No significant effect was demonstrated for anal sex without a condom or number of days on which drinking and sex occurred.	**Alcohol measures**: AUDIT, 90-day timeline follow-back (TLFB) at follow-up assessments. Differential loss to follow-up at 12 months (34% in intervention group and 26% in control group). Only 95 of 118 (81%) of the intervention group and 121 of 135 (90%) of the control group were included in the analyses.
Aharonovich et al. 2006	**Population** : 31 HIV-infected men and women engaged in HIV primary care. **Alcohol use**: All had four or more drinks at least once in the past 30 days, 55% had five or more drinks in the last week. **Setting** : HIV primary care clinic.	**Intervention**: 30-minute MI session on reducing alcohol use by counselor trained in MI plus an automated daily telephone self-monitoring interactive voice response (IVR) system with graphical feedback at 30-day follow-up meetings. **Control**: No control group. **Assessment:** Baseline, 30, 60, and 90 days.	**Drinks per day**: Using 7-day recall, mean drinks per day was 3.2 at baseline, 1.7 at 30 days, and 1.2 at 60 days. Mean highest drinks per day was 8.4, 4.1, and 3.8, respectively. **Cocaine use:** Decreased significantly at 60 days.	**Alcohol measures**: Quantity and frequency in past week and past month. Qualitative assessment of the program demonstrated satisfaction with daily calling and the feedback graph. Not a randomized controlled trial.
Parsons et al. 2007	**Population** : 143 HIV-infected subjects on antiretroviral therapy (ART) with hazardous drinking (more than 16 drinks per week for men, more than 12 drinks per week for women) recruited through HIV clinics and advertising from 2002 to 2005. **Setting** : Behavioral research center.	**Intervention** : RCT of eight 60-minute MI plus cognitive behavioral skills training (CBST) session by Masters-level counselors. **Control** : Eight 60-minute time and content-equivalent education sessions by health educators. All sessions delivered individually in private office over 12 weeks. **Assessment**: Baseline, 3 and 6 months.	**Alcohol use**: No significant effects on total drinks over 14 days or drinks per drinking day. Decreases in both groups from baseline to 3 and 6 months for these two drinking outcomes. **Medication adherence** : Significant improvement in dose and day adherence at 3 months, but difference not retained at 6 months. **HIV viral load/CD4 cell count**: Significant improvement at 3 months but not at 6 months.	**Alcohol measure** : Self-report 14-day TLFB to calculate total drinks and drinks per drinking day. **Adherence measures**: Self-report dose adherence = number of doses taken/number of doses scheduled over 14 days. Self-report dayadherence = number of days with perfect adherence/14 days.
Samet et al. 2005	**Population** : 151 HIV-infected patients on ART, with current or lifetime alcohol problems, determined by two or more positive responses on CAGE questionnaire or clinical diagnosis of alcohol disorder recruited from 1997 to 2000. **Setting**: Hospital (patients receiving HIV medical care).	**Intervention**: RCT of four 15-to 60-minute sessions over 3 months with MI-trained nurse who (1) addressed alcohol problems, (2) educated about ART efficacy, and (3) delivered tailored adherence advice including a reminder watch and a home visit. **Control:** Standard care **Assessment:** Baseline, 6, and 12 months.	**Alcohol use**: No significant effects on drinks per day, percent reporting any drinking, percent reporting hazardous drinking. **Medication adherence**: No significant effects on 3-day or 30-day adherence. **HIV viral load/ CD4 cell count**: No significant effects on mean CD4 cell count or mean log HIV RNA.	**Alcohol measures**: Self-report 30-day alcohol use from the Addiction Severity Index. **Adherence measures**: Self-reported AIDS Clinical Trial Group scale with 100% and 95% or more thresholds at 3-day and 30-day adherence, respectively.
**Category 2: Clinical trials among HIV-infected people of whom at least 10% have current alcohol use**				
Rotheram-Borus et al. 2009	**Population** : 270 HIV-infected people sexually active without a condom with at least one HIV-negative partner or two HIV-infected partners who were marginally housed and had four or more assessments; recruited from 2000 to 2002. **Alcohol use**: Mean number of days using alcohol or marijuana in the last 90 was 37. **Setting** : Recruited from community agencies, medical clinics, and advertisements.	**Intervention**: RCT of 15 90-minute individual counseling sessions, organized in three modules (“Coping” at 0–5 months, “Act Safe” at 5–10 months, and “Stay Healthy” at 10–15 months). **Control**: No intervention, only assessments **Assessment**: Baseline, 15, 20, and 25 months.	**Alcohol or marijuana use in the last 3 months** : At 25 months, the intervention group reduced its use from 36 to 28 days in the prior 90 days, whereas the control group was unchanged at 35 days of the last 90. Number of HIV negative partners and risky sexual acts also was reduced.	**Subanalysis of a clinical trial (Wong et al. 2008)**: 5% used alcohol/marijuana in the parent study. Proportion of alcohol users at baseline not presented in this study. Parent study reported only transmission act outcomes and demonstrated an effect that was not maintained at 25 months. Imbalance in transmission risk acts at baseline resulted in ineffective randomization, thus propensity scores were used to adjust for imbalances.
Naar-King et al. 2006, 2008	**Population**: 65 HIV-infected patients, aged 16–25 regardless of alcohol use or risk behaviors. **Alcohol use**: 77% lifetime, 39% had used alcohol in last 30 days at study entry. **Setting**: Adolescent HIV care clinic within a tertiary care children’s hospital.	**Intervention**: RCT of four 60-minute sessions of motivational enhancement. Therapy focused on two of three areas: substance use, sexual risk, or medication adherence over 10 weeks. **Control**: Wait list and standard care. **Assessment:** Baseline, 3, 6, and 9 months.	No significant effects at 9-month follow-up. **Alcohol use**: Borderline significant reduction in number of drinks in the week containing the maximum number of drinks (−9.65 vs. −1.3) at 3 months (*n* = 51). **Marijuana use**: Borderline significant reduction in number of times marijuana was used (*n* = 65). **Sexual risk**: Borderline significant reduction in total number of intercourse acts without a condom at 6 months (*n* = 65). **HIV viral load**: Significant reduction in log viral load at 6 months (*n* = 65).	**Alcohol and drug measures** : Timeline follow-back, though time window is not stated. **Sex risk measure** : Total number of unprotected intercourse acts without a condom. Note: 3-month outcomes on 51 subjects were published in 2006 and 6- and 9-month outcomes on 65 subjects published in 2008.
Gilbert et al. 2008	**Population**: 476 patients with alcohol risk (38%), defined as exceeding NIAAA safe drinking limits or drug risk (42%), or sex risk (60%), were recruited between 2003 and 2006. **Setting** : Outpatient HIV clinics.	**Intervention** : RCT of two sessions of tailored risk-reduction counseling at study entry and 3 months using a MI “Video Doctor” via laptop computer, printed educational worksheet, and delivery of a cueing sheet on reported risks to clinic care providers. **Control**: Standard care. **Assessment** : Baseline, 3, and 6 months.	**Alcohol use**: No significant effects on any risky drinking or number of drinks per week. **Drug use**: Significantly decreased 30-day illicit drug use at 3 and 6 months and fewer days of illicit drug use at 6 months. **Sex risk**: Significantly decreased 3-month unprotected sex at 3 and 6 months and fewer casual sex partners at 6 months. No effects on condom use.	**Alcohol measures**: Self-reported NIAAA risky drinking over 3 months. **Drug use measures** : Self-reported drug use over 30 days included any cocaine, methamphetamine, or heroin or 3 or more days of barbiturates, prescription opiates, hallucinogens, inhalants, or methylene-dioxymethamphetamine (MDMA).
Sorensen et al. 2003	**Population** : 190 HIV-infected patients with substance dependence; recruited from inpatient medical wards, detoxification clinic, and the emergency department from 1994 to 1996. **Alcohol use**: 61% in the last 30 days. **Setting**: Public general hospital.	**Intervention** : RCT of 12 months of case management by certified substance counselors in the community with caseload of 1:20 **Control**: Single brief contact with education about reducing HIV risk, information on HIV services, referrals to addiction treatment, social services. **Assessment**: Baseline, 6, 12, and 18 months.	No outcomes showed significant change between study groups at any time points, except decreased sex risk index. **Outcomes measured**: Addiction severity index composite scores, AIDS risk assessment scores, Beck depression inventory, health status questionnaire, and support evaluation list.	Summary/index score is shown without explanation of the raw measure.
Rotheram-Borus et al. 2001	**Population**: 310 HIV-infected patients (age 13–24) from nine adolescent clinics recruited from 1994 to 1996. **Alcohol use**: 67% nonabstinent at baseline. **Setting**: Adolescent clinics.	**Intervention** : 23 group sessions of two modules (“Stay Healthy” and “Act Safe”). **Control**: Standard care. Eligible for receiving the intervention at the conclusion of the study. **Assessment**: Baseline, 9, and 15 months.	**Alcohol/marijuana use:** 63% for attendees vs. 67% for control vs. 84% for nonattendees at 15 months.	Sequential assignment of 15 youths to intervention versus control groups (not randomized). The reported comparisons were attendees versus non-attendees versus control subjects. No intention-to-treat analysis was reported. Differential loss to follow-up. No alcohol-specific outcome was reported.
**Category 3: Randomized controlled trials among alcohol users at high risk for HIV infection**				
Morgenstern et al. 2007	**Population**: 198 MSM with current alcohol user disorder. **Alcohol use**: 88% with alcohol dependence. Mean drinks per drinking day was 10.4. **Setting** : Subjects recruited through advertisements in gay media, internet chat rooms, outreach to gay bars and clubs.	**Intervention**: 12 sessions of combined MI and coping skills training (MI+CBT) over 12 weeks (*n* = 47). **Control**: Four sessions of MI over 12 weeks (*n* = 42). Non–help-seeking (NHS) control group (*n* = 109). **Assessment**: Baseline, 12 weeks, and 12 months.	**Drinks per day**: At 12 weeks, the MI group had greater decreases in drinks per day than the MI+CBT group. This difference was not sustained at 12 months. Both intervention groups had greater decreases then the NHS group, but the NHS group also had substantial decreases in drinking.	**Alcohol measures**: CIDI at baseline. TLFB and short inventory of problems at followup. Potential subjects with drug use more severe than alcohol use disorder were excluded. Less than 10% HIV infected. Subjects lost to follow-up not included in the analysis.
Kalichman et al. 2008	**Population** : 342 men and women who drink in South African shebeens. **Setting**: Informal alcohol establishments (shebeens).	**Intervention**: 3-hour skills-based HIV–alcohol risk-reduction group session. **Control**: 1-hour HIV-alcohol information group session. **Assessment**: Baseline, 3, and 6 months.	The following behaviors were improved significantly at 3 months among the intervention group: alcohol use before sex unprotected intercourse percent of sex with condoms number of sex partners. Intervention effects were significantly stronger in those drinking less and dissipated at 6 months.	**Alcohol measures**: AUDIT, frequency of drinking before sex in previous month. Change in AUDIT scores not reported. 7% HIV infected in intervention group. 4% HIV infected in control group.

**Table 2:** Studies Identified but not Selected for the Literature Review

**Citation**	**Population**	**Reason Excluded**
Golin et al. 2003	140 HIV-infected patients. **Setting** : Hospital HIV clinic.	No data on the proportion of drinkers at baseline.
Goujard et al. 2003	326 HIV-infected patients. **Setting** : Hospital- and university-based centers.	No specific alcohol outcomes; alcohol group not analyzed independently.
Jones et al. 2003	174 women with AIDS from three U.S. cities recruited in 1997 from outpatient clinics, community health centers and agencies, and participant referrals. **Alcohol use**: 32% with history of alcohol. **Setting**: Primarily recruited from outpatient clinics, community health centers, and participant referrals.	No alcohol-specific outcomes reported.
Pradier et al. 2003	244 HAART-treated patients. **Setting** : Hospital	No specific alcohol outcomes; alcohol group not analyzed independently.
Samet et al. 2008	181 Russian men and women who reported any alcohol or drug dependence and who reported at least one incidence of unprotected sex in the past 6 months. **Setting**: Narcology hospitals	No alcohol-specific outcomes reported. Although both HIV-infected and alcohol-dependent patients were included in this study, the HIV-infected patients were not the alcohol-dependent patients.
Sampaio-Sa et al. 2008	107 HIV-infected, antiretroviral-naïve patients at an Brazilian HIV clinic for whom antiretrovirals were indicated were recruited from 2003 to 2004. 45% with alcohol use in the last 3 months.	Alcohol-specific outcomes not reported.
Simoni et al. 2007	136 HIV-infected men and women. **Setting:** Outpatient clinic	No information on current use; no specific alcohol outcomes.
Wong et al. 2008	936 HIV-infected from four U.S. cities recruited between 2000 and 2002. **Setting:** Community agencies, AIDS service organizations, and medical clinics	Alcohol-specific outcomes not reported; absolute numbers for outcome not presented.

**SOURCES**: Golin, C.E.; Earp, J.; Tien, H.C.; et al. A 2-arm, randomized, controlled trial of a motivational interviewing-based intervention to improve adherence to antiretroviral therapy (ART) among patients failing or initiating ART. *Journal of Acquired Immune Deficiency Syndromes* 42:42–51, 2006; Goujard, C.; Bernard, N.; Sohier, N.; et al. Impact of a patient education program on adherence to HIV medication: A randomized clinical trial. *Journal of Acquired Immune Deficiency Syndromes* 34:191–194, 2003; Jones, D.L.; Ishii, M.; LaPerriere, A.; et al. Influencing medication adherence among women with AIDS. *AIDS Care* 15:463–474, 2003; Pradier, C.; Bentz, L.; Spire, B.; et al. Efficacy of an educational and counseling intervention on adherence to highly active antiretroviral therapy: French prospective controlled study. *HIV Clinical Trials* 4:121–131, 2003; Samet, J.H.; Krupitsky, E.M.; Cheng, D.M.; et al. Mitigating risky sexual behaviors among Russian narcology hospital patients: The PREVENT (Partnership to Reduce the Epidemic Via Engagement in Narcology Treatment) randomized controlled trial. *Addiction* 103:1474–1483, 2008; Sampaio-Sa, M.; Page-Shafer, K.; Bangsberg, D.R.; et al. 100% adherence study: Educational workshops vs. video sessions to improve adherence among ART-naive patients in Salvador, Brazil. *AIDS and Behavior* 12:S54–S62, 2008; Simoni, J.M.; Pantalone, D.W.; Plummer, M.D.; and Huang, B. A randomized controlled trial of a peer support intervention targeting antiretroviral medication adherence and depressive symptomatology in HIV-positive men and women. *Health Psychology* 26:488–495, 2007; Wong, F.L.; Rotheram-Borus, M.J.; Lightfoot, M.; et al. Effects of behavioral intervention on substance use among people living with HIV: The Healthy Living Project randomized controlled study. *Addiction* 103:1206–1214, 2008.

## Footnotes

1 According to the National Institute on Alcohol Abuse and Alcoholism (2007), women who drink more than 3 drinks per day or more than 7 drinks per week and men who drink more than 4 drinks per day or more than 14 drinks per week are at increased risk for alcohol-related problems. Alcohol consumption levels above these limits are considered risky drinking.

2 RCTs are clinical studies in which patients randomly are assigned to either one or more groups receiving the treatment under investigation or to a control group receiving no treatment or a treatment of known efficacy.

3 The transtheoretical model (TTM) is a health behavior theory that assesses the individual’s readiness to change a particular behavior in order to facilitate the desired behavior change. The stages of change are: precontemplation, contemplation, preparation, action, and maintenance.

4 With a time- and content-equivalent control group, participants in that group spend the same amount of time with a health care provider/therapist as the intervention group, and they receive the same type of information. The only difference between the intervention and control groups is the method used to deliver the information, allowing researchers to determine whether one approach is more effective than the other.

5 CD4 cells are a type of white blood cell that is the main target of the HIV virus; accordingly, levels of these cells in the blood decline with progressing HIV infection and are a marker for disease progression.

6 An intention-to-treat analysis is based on the initial treatment intent, not on the treatment actually administered. Thus, every participant who begins the treatment is considered to be part of the trial, whether they finish it or not. This is done to avoid various misleading artifacts that can arise in a study. For example, if participants who have a more serious problem tend to drop out at a higher rate, even an ineffective treatment may appear to provide benefits if one only compares the condition before and after the treatment among participants who finish the treatment and ignores participants who were enrolled originally but did not finish the treatment.
